# An Alignment-Independent 3D-QSAR Study of FGFR2 Tyrosine Kinase Inhibitors

**DOI:** 10.15171/apb.2017.049

**Published:** 2017-09-25

**Authors:** Behzad Jafari, Maryam Hamzeh-Mivehroud, Ali Akbar Alizadeh, Mehdi Sharifi, Siavoush Dastmalchi

**Affiliations:** ^1^Biotechnology Research Center, Tabriz University of Medical Sciences, Tabriz, Iran.; ^2^School of Pharmacy, Tabriz University of Medical Sciences, Tabriz, Iran.; ^3^Students Research Committee, Tabriz University of Medical Sciences, Tabriz, Iran.

**Keywords:** 3D-QSAR, Docking, GRIND descriptors, Tyrosine kinase inhibitors, FGFR2

## Abstract

***Purpose:*** Receptor tyrosine kinase (RTK) inhibitors are widely used pharmaceuticals in cancer therapy. Fibroblast growth factor receptors (FGFRs) are members of RTK superfamily which are highly expressed on the surface of carcinoma associate fibroblasts (CAFs). The involvement of FGFRs in different types of cancer makes them promising target in cancer therapy and hence, the identification of novel FGFR inhibitors is of great interest. In the current study we aimed to develop an alignment independent three dimensional quantitative structure-activity relationship (3D-QSAR) model for a set of 26 FGFR2 kinase inhibitors allowing the prediction of activity and identification of important structural features for these inhibitors.

***Methods:*** Pentacle software was used to calculate grid independent descriptors (GRIND) for the active conformers generated by docking followed by the selection of significant variables using fractional factorial design (FFD). The partial least squares (PLS) model generated based on the remaining descriptors was assessed by internal and external validation methods.

***Results:*** Six variables were identified as the most important probes-interacting descriptors with high impact on the biological activity of the compounds. Internal and external validations were lead to good statistical parameters (r^2^ values of 0.93 and 0.665, respectively).

***Conclusion:*** The results showed that the model has good predictive power and may be used for designing novel FGFR2 inhibitors.

## Introduction

It is well known that the interaction between different components of tumor microenvironment play crucial role in progression and malignancy of the tumor.^1^ Among the cells present in the turmeric area, fibroblasts were gained much attention due to having distinguished characteristics compared with fibroblasts in normal tissues. Such fibroblasts in turmeric area are termed carcinoma associate fibroblasts (CAFs) and are detectable in various tumors including breast, prostate, lung, colon and pancreas cancers.^2^

Fibroblast growth factor receptors (FGFRs) presented on the surface of CAFs are belong to the transmembrane receptors known as receptor tyrosine kinases (RTKs) comprised of three immunoglobulin-like domains at the extracellular region connected via a single transmembrane region to the intracellular tyrosine kinase domain.^3^ FGFR family consist of four closely related receptors called FGFR1 to FGFR4. Ligand-activated FGFRs activate signaling pathways in the cell which lead to cell proliferation, growth, differentiation, migration, and survival.^4^ Similar to other RTKs, deregulation of these receptors can trigger numerous diseases including cancer. FGFR2 as one of the important factors on the surface of CAFs is overexpressed in some human cancers including stomach, pancreas, and breast. Moreover, mutations of this receptor can lead to intrinsically active form of FGFR2 reported in endometrial and lung cancers.^5^ Inhibition of RTKs as a promising target in treatment of different kinds of cancers has been led to development of remarkable therapeutic agents.^6^ Most of these tyrosine kinase inhibitors (TKI) at different clinical phases are the small molecules targeting ATP-binding site of the kinase domain of RTKs.^7^

In the context of developing new therapeutics, high-throughput studies combined with computational analyses are effective tools for lead compound discovery.^8^ Quantitative structure-activity relationship (QSAR) is one of the most commonly *in silico* methods for the prediction of biological activity of compounds by transforming their chemical and structural properties into numerical values which can then be linked to their potencies using mathematical models.^9^ There are different types of QSAR from dimensionality point of view of which 3D-QSAR method is extensively used in drug design and discovery process. In this methodology, 3D-descriptors which are representative of atomic arrangement in 3D space are employed to be used in alignment-dependent or alignment free analyses. In alignment-dependent analysis the studied compounds are required to be aligned with each other whereas in alignment free method there is no need for superpositioning of molecules prior to development of 3D models, which can be considered as an advantage. GRid-Independent Descriptors (GRIND) is one of the alignment-independent methods^10,11^ in which molecular interaction fields (MIF) are used to describe the interaction energy between ligands and different types of probes.^12^ Then data mining are performed on the pool of calculated descriptors according to their impact on the biological activity followed by calculating favorable and unfavorable interactions.^13^

In the current study we aimed to develop a 3D-QSAR model using GRIND algorithm for a set of FGFR2 kinase inhibitors to identify the needed structural requirements. The results of the current study may be used for designing novel FGFR2 inhibitors.


Table 1. Structures and biological activities of FGFR2 inhibitors

**Figure:**
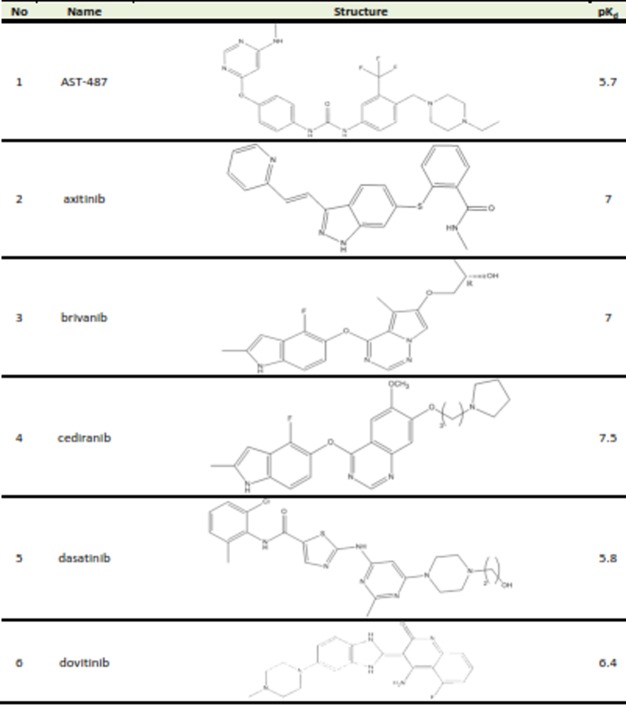

**Figure:**
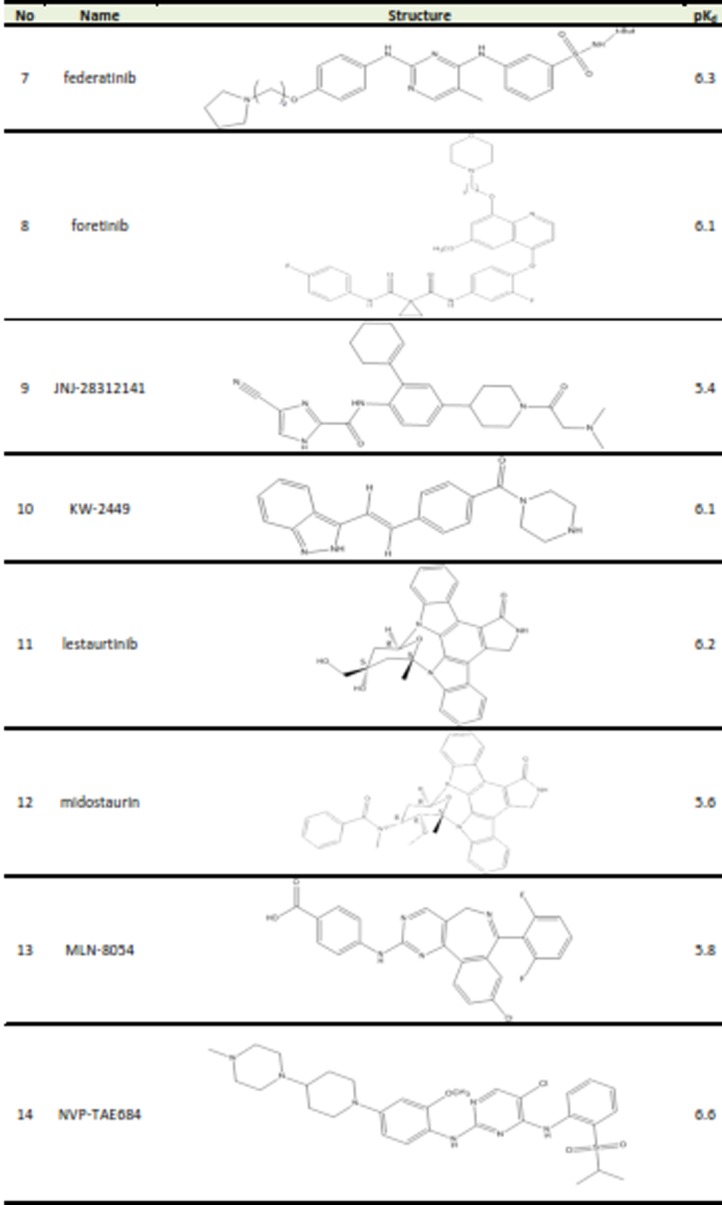

**Figure:**
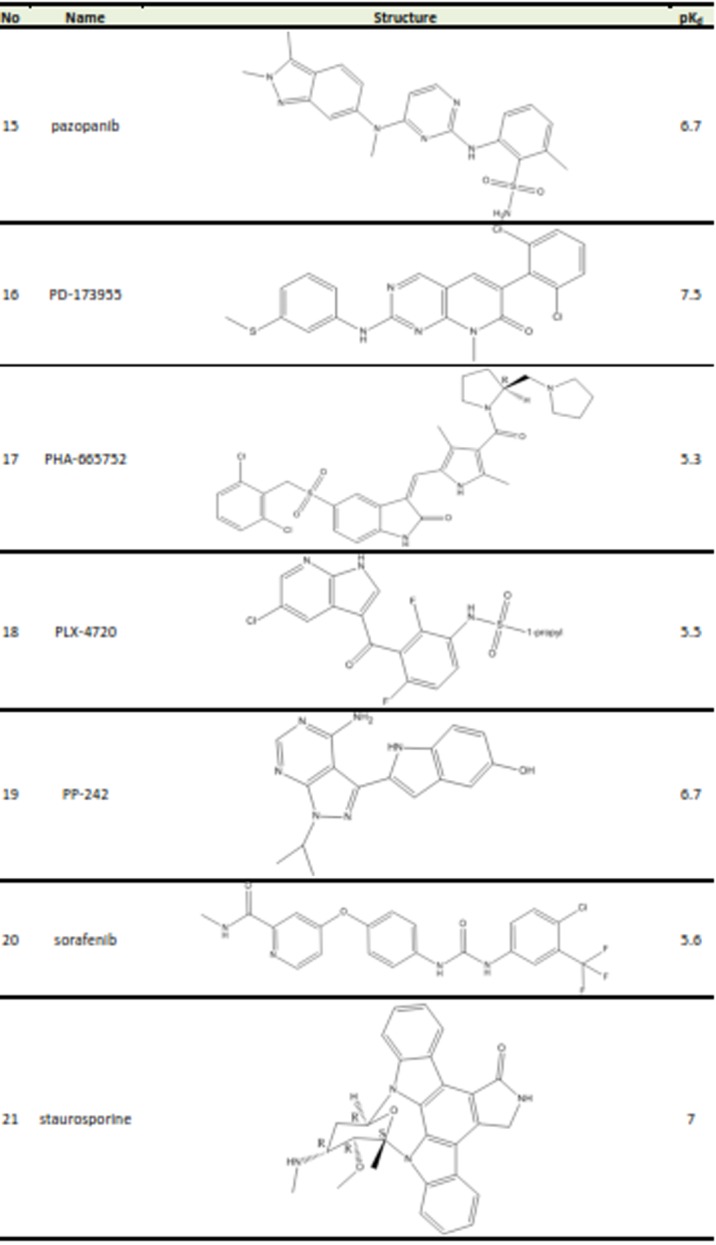

**Figure:**
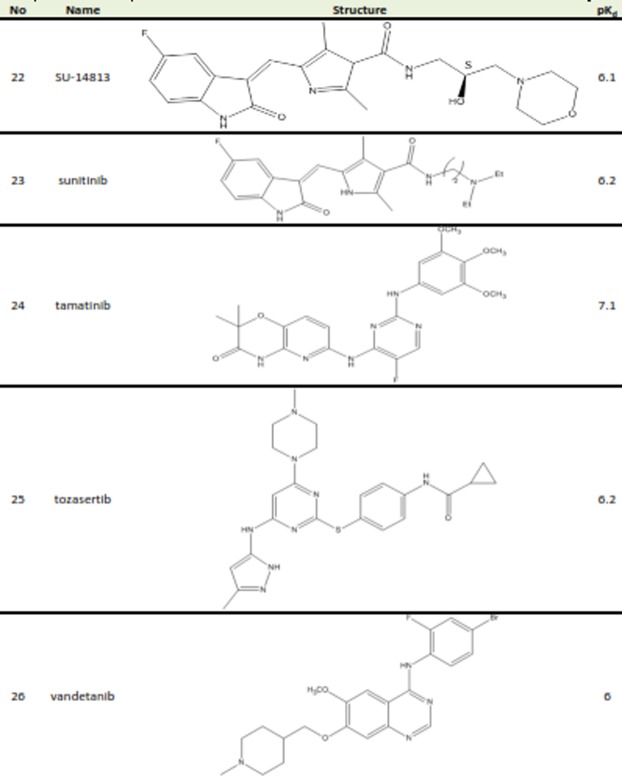

## Materials and Methods

### Data set preparation

A set of 26 small molecules with inhibitory activity on FGFR2 were collected from the literature.^14-16^ Inhibitory activities of the studied compounds reported in Kd (nM) were converted to pK_d_ values. The transformed data would be used as dependent variable in 3D-QSAR study. Table 1 presents the structures and corresponding pK_d_ values of FGFR2 kinase inhibitors. The 3D structures of the molecules were generated using the Built Optimum option of Hyperchem software (version 8.0.8) followed by energy minimization using MM+ force field based on Polack‐Ribiere algorithm.^17^ Then, the structures were fully optimized based on the semiemperical method at AM1 level of theory.^18^

### Molecular docking study

The crystal structure of kinase domains for FGFR1 and FGFR2 (PDB IDs: 5A46 and 3RI1 respectively) were retrieved from Protein Data Bank. Docking analysis of the energy-minimized compounds was performed using AutoDock software version 4.2^19^ running under LINUX operating system. The binding site was determined based on position of co-crystallized inhibitor compound. AutoGrid was used for the preparation of the grid map using a grid box. The grid size was set to 40 × 40 × 40 xyz points with grid spacing of 0.375 Å and the box was centered at point with -15.788, 23.568, and -33.739 (x, y, and z) coordinates. For docking experiment, Lamarckian genetic algorithm (LGA) was employed in a way that the number of generation, energy evaluations, and individuals in the population were set to 27000, 2.5× 10^6^,and 150, respectively. The number of docking solutions was set to 100, and the default values were accepted for the rest of parameters.

### Calculation of GRIND descriptors and model building

The docking solutions for each compound were filtered based on the similarity to the reference structure (i.e. dovitinib) using Shape-it™ software.^20^ The selected conformers were introduced to Pentacle program to generate GRIND-based descriptors. To do this, first MIFs were generated using GRID-based fields in which the interaction energies between atoms of molecules and different probes including hydrophobic (DRY), hydrogen bond donor, HBD (O), hydrogen bond acceptor, HBA (N1), and shape (TIP) probes at the given cutoff distance are calculated. The interaction energies (E_xyz_) at each grid point called node were the sum of Lennard-Jones energy (E_lj_), hydrogen bond (E_hb_), and electrostatic interactions (E_el_). Based on the defined cutoff, the nodes having energies lower than the cutoff were discarded. To this end, ALMOND algorithm was employed to extract the most relevant regions from MIFs according to the field intensity at a node and the mutual node-node distances between the selected nodes. Finally‏, MIFs were encoded by maximum autocorrelation and cross-correlation algorithm for generating correlograms in which the product of node-node energies were plotted *vs* the distances between the nodes.

### Modeling and statistical analyses

The entire dataset was randomly divided into training and test sets containing 21 and 5, compounds, respectively. For generating 3D-QSAR model, fractional factorial design (FFD), was applied on training subset of data for obtaining the descriptors explaining the important interactions with defined probes. FFD method was carried out until no significant change in the model statistical parameters such as r^2^ and q^2^ was observed. The remaining descriptors were subjected to partial least squares (PLS) regression where the descriptors internally cross-validated using leave-one-out (LOO), leave-two-out (LTO), and random-group-out (RGO). The PLS model was also externally evaluated with 5 randomly selected test set compounds. To further evaluate the robustness of the generated model, *y-scrambling* test was carried out by ten times randomly scrambling the activity data for the train set and generating PLS models as outlined above. The generated PLS models were utilized to predict the activity of test set compounds.

## Results and Discussion

**Figure 1:**
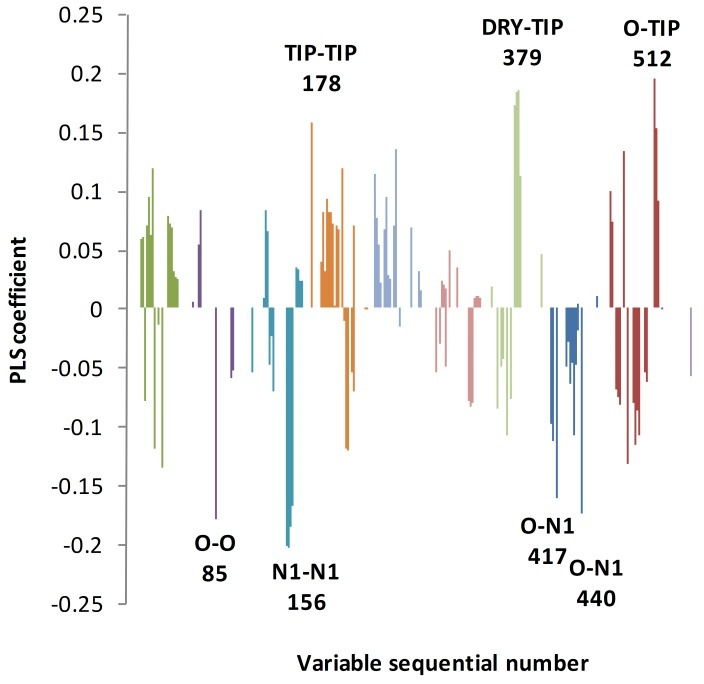
2LV PLS coefficient plots for the obtained model. The most intensive variables are labeled by sequential numbers. 2LV indicates 2 latent variables; PLS, partial least squares.

Targeting carcinoma associate fibroblasts (CAFs) as one of the important components presented in the microenvironment of turmeric area was the focus of many research activities recently.^1,2,21,22^ FGFR proteins as the cell surface elements of CAFs interact with their endogenous ligands and interfere with these interactions by small molecule inhibitors is one of the promising strategies which may lead to the development of new anticancer agents.^4^ In general, the drug development processes require extensive experimental studies to find and improve the potency and pharmacokinetics of drug candidates via optimizing their 3D structures and physicochemical properties. Identification of novel TKIs are no exception and variety of technologies such as high-throughput screening are being employed extensively to develop druggable compounds acting as tyrosine kinase inhibitor.^23^ The highly expensive and time-consuming procedure of drug development requires the utilization of complementary *in silico* methods to cut-down the cost and time and increase the success rate.^11^ In the current study we have used *in silico* QSAR approach based on alignment independent method to generate a 3D model for activity prediction of a set of FGFR2 inhibitors. One of the important issues in alignment independent 3D-QSAR calculations is the use of active conformation of the studied compounds. To consider an appropriate measure regarding this criterion, the best fitting conformers of the compounds to the reference structure dovitinib were selected after docking procedure. The experimental data showing the receptor bound form of reference compound (FGFR2- dovitinib complex) is not available, however, its crystal structure in complex with TK domain of closely homologous receptor FGFR1 has been reported previously.^24^ By superpositioning the crystal structures of TK domains of FGFR1 and 2, the bound conformation of dovitinib at the TK domain of FGFR2 was identified and subsequently was used as the reference structure to filter the docking results. The filtered conformations for the TK inhibitors were submitted to Pentacle software for alignment independent 3D-QSAR analyses. FFD feature selection was applied to select important independent variables calculated by the software in relation to the dependent variable pK_d_. Based on the remained variables PLS model was generated. Figure 1 represents PLS coefficients of variables which were selected by applying FFD on the GRIND descriptors using Pentacle software. The important descriptors based on their corresponding PLS coefficients were listed in Table 2. According to Table 2 two descriptors have positive impact on the biological activity of the compounds while four of them have negative impact.

**Table 2:** The most important structural variables in the 3D-QSAR model

**Probes**	**Distance (Å)**	**Variable**	**Impact**	**Expression**	**Element**
O-O	10.8-11.2	85	negative	about half of the compounds	NH of the amide in the ring or outside the ring and NH or its bioisoesters
O-N1	13.6-14	440	negative	almost all of the compounds	amine of indole/ piperazine/ pyrrole piperidine/ pyridine/ quinolone and hydroxyl /amine of endmost moiety or NH between two heterocyclic ring or indole ring
O-N1	4.4-4.8	417	negative	almost all of the compounds	amide carbonyl/ sulfonamide nitrogen and oh of endmost moiety/ NH of amide or NH in the indole ring
O-TIP	19.2-19.6	512	positive	one third of the compounds	NH in the ring or amide and endmost heterocyclic ring/methyl attached to the indole ring
DRY-TIP	12.2-12.8	379	positive	all of the compounds	indole/quinolone ring (or bioisoesters) and halide/alkyl in the end most moiety
N1-N1	16-16.4	156	negative	one third of the compounds	nitrogen of the heterocyclic compounds (piperazine and indole ring) and nitrogen of amide

Variable 379 belonging to DRY-TIP block with positive impact on biological activity has been observed for all of the compounds. The higher the activity of the compound is, the higher the DRY-TIP values. In this variable, DRY as a hydrophobic probe interacts with indole or quinolone rings or their corresponding bioisosteres and is connected to TIP interacting moieties such as halides or alkyl groups at the endmost part of the compounds separated with a distance ranging from 12.2 to 12.8 Å. In compounds 10, 11, and 12, with more rigid structures, the TIP interacting groups are indole or indazole groups. The quantity of DRY-TIP variable for the reference compound dovitinib (structure 6 in this study) in the receptor bound conformation is in good agreement with its potency (i.e., pK_d_ value), validating the statement made regarding the importance of this variable. Moreover, the inspection of experimental dovitinib-FGFR1 structure shows that the DRY-TIP interacting structural components are involved in interactions with Leu^630^, Val^492^, and Gly^567^ residues in kinase domain of FGFR1.^24^ Another important cross correlogram with positive impact on the activity is O-TIP, which relates NH of ring or amide groups with hot spot part at the far end of heterocyclic ring or methyl group attached to indole ring with a distance ranging from 19.2-19.6Å (variable 512). This is also in close agreement with the crystal structure (PDB ID: 4AGD)^25^ of VEGF receptor (VEGFR) in complex with Sunitinib (structure 23). Sunitinib is able to interact via NH of its indole ring with Glu^917^ of VEGFR.

Another important variable negatively influencing the activity is auto-corralogram O-O (variable 85). This variable indicates that the presence of two hydrogen bond donor groups on the compounds separated by 10.8-11.2Å is not favorable. These H-bond donor groups with the relative distances identified in variable 85 are presented by NH or OH moieties, and are shown just in less potent compounds. Thakur et al. has solved the structure of dasatinib (compound 5) bound to human protein tyrosine kinase 6 (PDB ID 5H2U) and identified a hydrogen bond interaction between its NH group positioned between two heterocyclic rings with Met^267^ residue.^26^ However, the second H-bond donor group is not participated in any H-bond interaction, which may suggest an unfavorable effect for the presence of latter group at the defined distance from the former group.

Variables 440 and 417 belonging to O-N1 cross-correlogram are among the informative variables, which have negative impact on the inhibitory activity of the compounds. These variables are expressed by all compounds in the dataset. The effect of descriptor 417 is more pronounced for less potent compounds. The structure-activity relationship (SAR) study on some dovitinib analogues showed that the presence of a hydrogen bond donor-acceptor such as hydroxyl or amine group is important for FGFR1 TK inhibitory activity.^27^ In dovitinib this H-bond donor-acceptor group is the NH2 moiety acting as the H-bond acceptor characteristics of the O-N1 variable, which seems to have negative effect on inhibitory activity. Apparently, this is in contrast to the results of SAR studies reported by Renhowe et al., who found this group as a necessary structural feature for having TK inhibitory activity. The source of such seemingly clear disagreement may be the huge differences in the chemical structures of the compounds used in this study with theirs all being close analogues of dovitinib. Apart from this, the compounds lacking the H-bond donor-acceptor group in Renhowe study are either inactive or very less active, while all compounds in this study are active and the unfavorable effect attributed to the N1 interacting group participating in O-N1 variable shows only its relative effect in this dataset. Moreover, the biological activity values are measured using TKs from different receptors (FGFR1 and 2), which makes comparison more difficult. In contrast to the variable 417, O-N1 variable 440 includes wide variety of structural moieties and shows less linear correlation to the activity.

**Figure 2:**
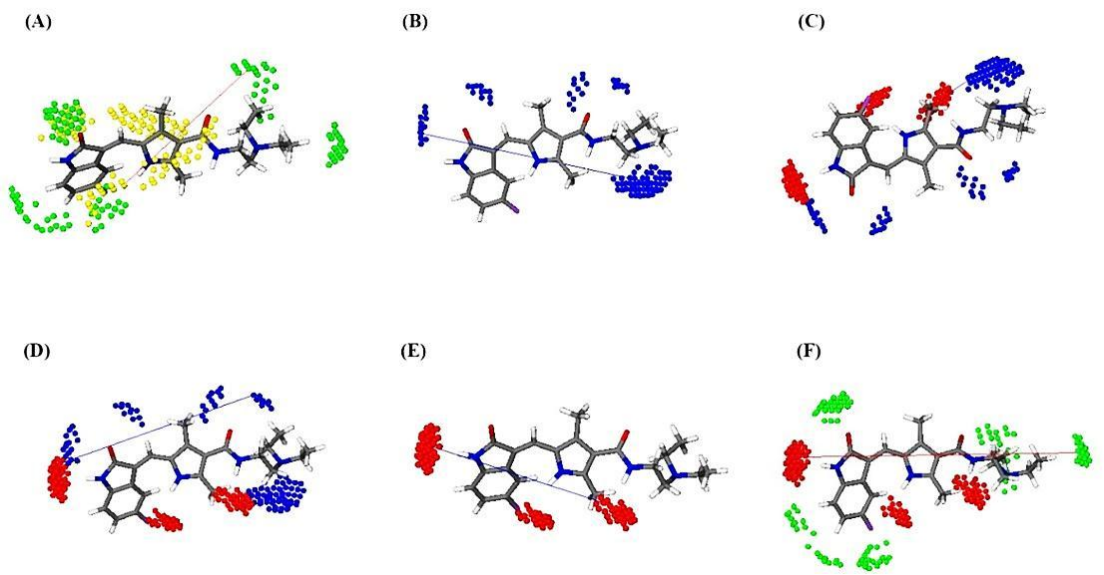
The most important structural elements associated with variables: (a) DRY‐TIP 379; (b) N1‐N1 156; (c) O-N1 417; (d) O-N1 440; (e) O-O 85; (f) O-TIP 512

At last but not least, the last selected variable 156 is of N1-N1 block with the highest negative impact on the biological activity. This descriptor is observed only in weaker compounds implying that the presence of N1-N1 interacting groups at the distance of 16-16.4 Å unfavors TK inhibition. Figure 2 represents 6 probe interaction blocks for compound 23.

Considering that the study was carried out using 26 inhibitors, only 5 compounds (20% of data set) were randomly selected as the test set and the remaining 21 compounds were used to develop the model. Using bigger test set would have adversely affected the predictivity of the model due to loss of information as the result of developing the model by fewer training set compounds. Table 3 shows the statistics for the PLS model generated using training set FGFR2 inhibitors for three latent variables (3LV). Internal validations with leave-one-out (LOO), leave-two-out (LTO), and five random-group-out (RGO) methods were performed to assess robustness of the model. The results suggested a significant correlation between PLS components and FGFR2 inhibitory activities of the compounds. In order to assess the predictive performance of the model, a subset of 5 randomly selected molecules as the test set (shown in Table 4) were used for the prediction. By monitoring the changes in the statistical indices (shown in Table 3), two latent variables (2LVs) were selected as the optimum number of PLS components for the model interpretation.

**Table 3:** Statistics for the PLS model for FGFR2 inhibitors

**LV**	**SSX**	**SSX**_ACC_	**SDEC**	**SDEP**	**r** ^ 2 ^	**r** ^ 2 ^_ACC_	**q** ^ 2 ^_ACC_ **(LOO)**	**q** ^ 2 ^_ACC_ **(LTO)**	**q** ^ 2 ^_ACC_ **(5RG)**
1	10.55	10.55	0.21	0.38	0.87	0.87	0.59	0.55	0.49
2	10.76	21.31	0.15	0.29	0.06	0.93	0.75	0.71	0.65
3	10.16	31.47	0.11	0.29	0.03	0.96	0.76	0.75	0.67

**Table 4:** Observed vs predicted inhibitory activities for FGFR2 inhibitors used in this work

**Comp**	**pK**_d_ ** (exp)**	**pK**_d_ ** (pred)**	**Comp**	**pK**_d_ ** (exp)**	**pK**_d_ ** (pred)**
1	5.7	5.77	14	6.6	6.48
2	7	7.06	15	6.7	6.85
3	7	7.04	16^a^	7.5	6.79
4	7.5	7.65	17	5.3	5.41
5	5.8	5.87	18	5.5	5.48
6	6.4	6.37	19^a^	6.7	6.63
7^a^	6.3	6.53	20	5.6	5.65
8	6.1	6.03	21	7	6.82
9^a^	5.4	5.95	22	6.1	6.00
10	6.1	5.95	23	6.2	6.13
11	6.2	6.29	24	7.1	6.92
12	5.6	5.82	25	6.2	6.20
13^a^	5.8	5.59	26	6	5.91

^a^Test set compounds.

**Figure 3:**
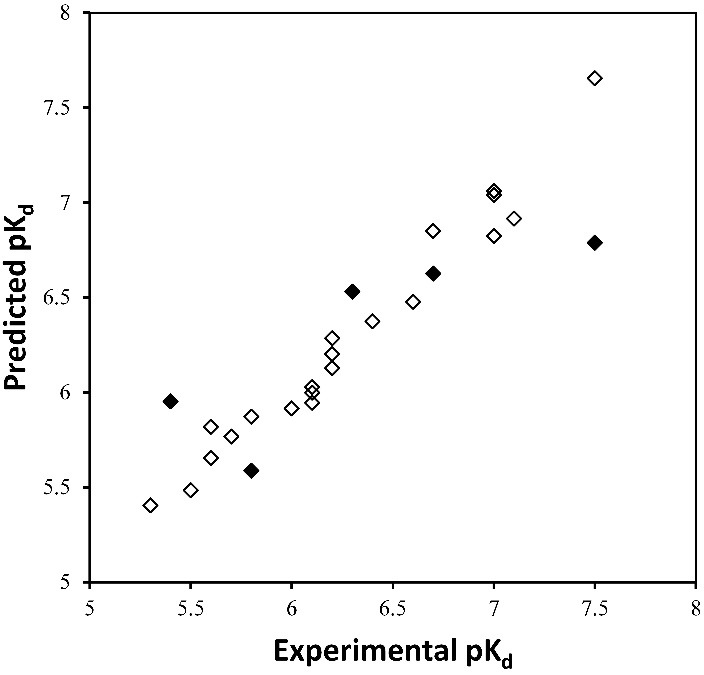
Experimental vs predicted pK_d_ for compounds. Open squares indicate training set and filled circles show the test set compounds

Figure 3 presents experimental value of pK_d_ against predicted activities for the training and test sets. External validation assessment showed a good predictivity for the model with r^2^of 0.665 and SDEP of 0.29 measured for the test compounds. For further validation of the model, ten *y-randomization* tests were performed by randomly scrambling the biological activities for each data point. The results showed that the average squared correlation coefficient (r^2^) was -0.292 indicating that the model was not developed by chance. Another validity criterion measured for the developed model was the percentage of prediction errors. A useful rule of thumb is that a prediction error for the test set compounds smaller than or equal to 10% of the training set activity range should be considered acceptable, while an error value greater than 20% is considered a high percentage error. The calculated mean absolute error for the test set compounds was 0.35 (equal to 15% of training set activity range) which is greater than 10% of the activity range for the training set compounds. But, it is less than 20% of the range, which collectively considering the results of all validation assessments seems reasonable.^28^ The applicability domain was defined according to the method developed by Roy *et al*, termed applicability domain using standardization approach.^29^ For this purpose PLS latent variables obtained from 3D-QSAR model and pK_d_ values were used as X and Y variables, respectively. The result showed that there are no outliers among the dataset compounds. Hence the model can be applied for the prediction of FGFR2 tyrosine kinase inhibitory activity of compounds having structures similar to those used in this study. Taken together, working with small data set has some challenging issues concerning external validation. However, this limitation can be overcome by providing more data obtained from experimental procedures.

## Conclusion

In summary, an alignment independent 3D-QSAR model was generated for a set of tyrosine kinase inhibitors (TKIs) evaluated on FGFR2. The model was verified with internal and external validation methods as well as *y-scrambling* technique. The results showed that the model has good predictive power with acceptable statistics. According to the selected correlograms, indole and quinolone rings as well as their bioisosteres are important moieties in the structures. These structural features are involved in hydrogen bond and hydrophobic interactions. Moreover, in some compounds substitution of these moieties with small heterocyclic groups helps to retain the functionality of the compounds. The result of the current investigation can be used in designing the novel FGFR2 tyrosine kinase inhibitors.

## Acknowledgments

The authors would like to thank the Research Office and Biotechnology Research Center of Tabriz University of Medical Sciences for providing financial support under the Postgraduate Research Grant scheme for the PhD thesis of BJ.

## Ethical Issues

Not applicable.

## Conflict of Interest

The authors declare no conflict of interests.
